# Transcatheter Aortic Valve Replacement with Self-Expandable ACURATE neo as compared to Balloon-Expandable SAPIEN 3 in Patients with Severe Aortic Stenosis: Meta-analysis of Randomized and Propensity-Matched Studies. *J. Clin. Med.* 2020, *9*, 397

**DOI:** 10.3390/jcm9030861

**Published:** 2020-03-20

**Authors:** Mirosław Gozdek, Kamil Zieliński, Michał Pasierski, Matteo Matteucci, Dario Fina, Federica Jiritano, Paolo Meani, Giuseppe Maria Raffa, Pietro Giorgio Malvindi, Michele Pilato, Domenico Paparella, Artur Słomka, Jacek Kubica, Dariusz Jagielak, Roberto Lorusso, Piotr Suwalski, Mariusz Kowalewski

**Affiliations:** 1 Department of Cardiology and Internal Medicine, Nicolaus Copernicus University, Collegium Medicum, 85067 Bydgoszcz, Poland; gozdekm@wp.pl (M.G.); kubicajw@gmail.com (J.K.); 2 Thoracic Research Centre, Nicolaus Copernicus University, Collegium Medicum in Bydgoszcz, Innovative Medical Forum, 85067 Bydgoszcz, Poland; kamilziel@gmail.com (K.Z.); michalpasierski@gmail.com (M.P.); artur.slomka@cm.umk.pl (A.S.); 3 Department of Cardiology, Warsaw Medical University, 02091 Warsaw, Poland; 4 Clinical Department of Cardiac Surgery, Central Clinical Hospital of the Ministry of Interior and Administration, Centre of Postgraduate Medical Education, 02607 Warsaw, Poland; suwalski.piotr@gmail.com; 5 Department of Cardio-Thoracic Surgery, Heart and Vascular Centre, Maastricht University Medical Centre, 6229 HX Maastricht, The Netherlands; teo.matte@libero.it (M.M.); dario.fina88@gmail.com (D.F.); fede.j@hotmail.it (F.J.); paolo.meani@ospedaleniguarda.it (P.M.); roberto.lorussobs@gmail.com (R.L.); 6 Department of Cardiac Surgery, Circolo Hospital, University of Insubria, 21100 Varese, Italy; 7 Department of Cardiology, IRCCS Policlinico San Donato, University of Milan, 20097 Milan, Italy; 8 Department of Cardiac Surgery, University Magna Graecia of Catanzaro, 88100 Catanzaro, Italy; 9 Department of Intensive Care Unit, Maastricht University Medical Centre (MUMC+), 6229 HX Maastricht, The Netherlands; 10 Department for the Treatment and Study of Cardiothoracic Diseases and Cardiothoracic Transplantation, IRCCS-ISMETT (Instituto Mediterraneo per i Trapianti e Terapie ad alta specializzazione), 90127 Palermo, Italy, mpilato@ISMETT.edu (M.P.); 11 Wessex Cardiothoracic Centre, University Hospital Southampton, Southampton SO16 6YD, UK; pg.malvindi@hotmail.com; 12 GVM Care & Research, Department of Cardiovascular Surgery, Santa Maria Hospital, 70124 Bari, Italy; domenico.paparella@uniba.it; 13 Department of Emergency and Organ Transplant, University of Bari Aldo Moro, 70121 Bari, Italy; 14 Chair and Department of Pathophysiology, Nicolaus Copernicus University, Collegium Medicum, 85067 Bydgoszcz, Poland; 15 Department of Cardiac Surgery, Gdańsk Medical University, 80210 Gdańsk, Poland; kardchir@gumed.edu.pl

The authors sincerely apologise for the imperfections made during the collection of the data and wish to make the following corrections to this paper [1].

(1) Abstract 

ACURATE neo was associated with a 3.7-fold increase in moderate-to-severe PVL (RR (risk ratio): 3.70 (2.04–6.70); P < 0.0001), which was indirectly related to higher observed 30-day mortality with ACURATE valve (RR: 1.77 (1.03–3.04); *P* = 0.04).

Should be replaced with

ACURATE neo was associated with an over 3-fold increase in moderate-to-severe PVL (RR (risk ratio): 3.06 [2.09–4.49]; *P* < 0.00001), which was related to higher observed 30-day mortality with ACURATE valve (RR: 1.77 (1.03–3.04); *P* = 0.04).

(2) Abstract 

In conclusion, ACURATE neo, as compared with SAPIEN 3, was associated with higher rates of moderate-to-severe PVL, which were indirectly linked with increased observed 30-day all-cause mortality.

Should be replaced with

In conclusion, ACURATE neo, as compared with SAPIEN 3, was associated with higher rates of moderate-to-severe PVL, which were further linked to increased observed 30-day all-cause mortality.

(3) 3.3. Procedural Outcomes

**Figure A4:**
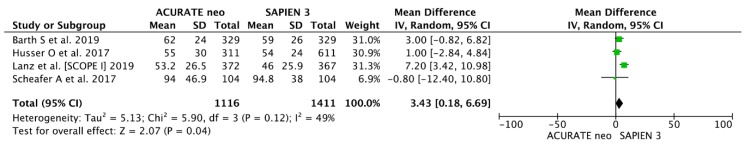
Procedural outcomes. Detailed analysis of individual weighted mean differences (MDs) with corresponding 95% CIs on procedure duration for the comparison of ACURATE neo vs. SAPIEN 3.

Four studies [14,15,17,19] including 1116 ACURATE neo and 1411 SAPIEN 3 cases provided data on procedure duration, which was, on average, 3 minutes longer in the former: 60.1 ± 28.6 min. vs. 56.5.9 ± 26.0 min. (MD 3.06, 95% CI, (−0.66, 6.76) min) without reaching statistical significance (Figure A4).

Should be replaced with

Four studies [14,15,17,19] including 1116 ACURATE neo and 1411 SAPIEN 3 cases provided data on procedure duration, which, on average, took 3.5 minutes longer in the former: 60.1 ± 28.6 min. vs. 56.1 ± 26.0 min. (MD 3.43, 95% CI, (0.18, 6.69) min) (Figure A4).

(4) 3.4. Clinical Outcomes

**Figure 3:**
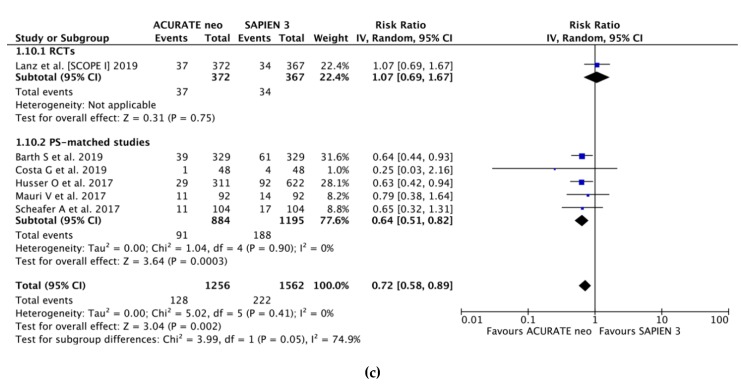
Individual and summary risk ratios with corresponding 95% confidence intervals for the comparison of ACURATE neo vs. SAPIEN 3 in the analysis of clinical outcomes: (**a**) early safety, (**b**)·device success and (**c**) permanent pacemaker implantation.

Based on the data from six studies (2818 pts.), PPI was required nearly 30% less often after ACURATE neo implantation as compared to SAPIEN 3 (RR 0.72, 95% CI, (0.58, 0.89); *P* = 0.003; I2 = 75.9%) with corresponding frequency of 10.1% vs. 14.2%, respectively (Figure 3c). Importantly, the estimates derived from SCOPE I differed from the pooled estimates (P_interaction_ = 0.04) with higher rates of PPI observed in SAPIEN 3 arm in PS-matched studies (9.3% vs. 15.8%).

Should be replaced with

Based on the data from six studies (2818 pts.), PPI was required nearly 30% less often after ACURATE neo implantation as compared to SAPIEN 3 (RR 0.72, 95% CI, (0.58, 0.89); *P* = 0.002; I2 = 0%) with corresponding frequency of 10.2% vs. 14.2%, respectively (Figure 3c). Importantly, the estimates derived from SCOPE I differed from the pooled estimates (P_interaction_ = 0.05), with higher rates of PPI observed in SAPIEN 3 arm in PS-matched studies (9.3% vs. 15.8%).

(5) 3.5. Functional Outcomes

**Figure 4:**
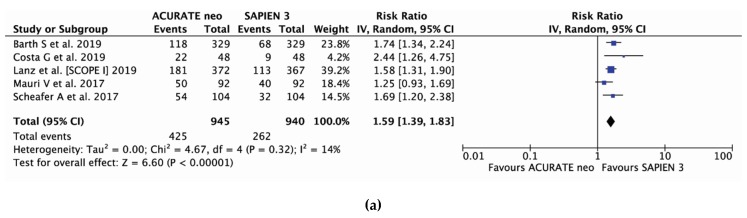

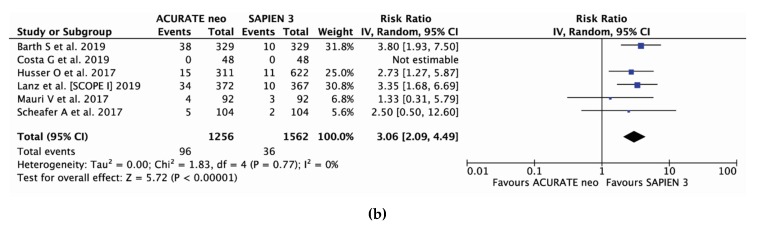
Individual and summary risk ratios with corresponding 95% confidence intervals for the comparison of ACURATE neo vs. SAPIEN 3 in the analysis of functional outcomes: (**a**) mild and (**b**) moderate-to-severe paravalvular leak.

With five studies [14–16,18,19] and 1885 patients included, mild PVL occurred less frequently in SAPIEN 3 recipients, 28.0% (263 of 940), compared to ACURATE neo group, 45.5% (430 of 945); (RR 1.60, 95% CI, (1.40, 1.84) *P* < 0.00001; I2 = 14%) (Figure 4a). Moderate-to-severe PVL was uncommon in the entire series (6.5%); however, there was a significant 3.7-fold increase in moderate-to-severe PVL risk with ACURATE neo implantation: (RR 3.70, 95% CI, (2.04, 6.70) *P* < 0.0001; I2 = 53%) (Figure 4b) and corresponding incidence of 11.7% (147/1,256) and 2.3% (36/1,562) in ACURATE neo and SAPIEN 3 valves.

Should be replaced with

With five studies [14–16,18,19] and 1885 patients included, mild PVL occurred less frequently in SAPIEN 3 recipients, 27.9% (262 of 940), compared to ACURATE neo group, 45.0% (425 of 945); (RR 1.59, 95% CI, (1.39, 1.83) *P* < 0.00001; I^2^ = 14%) (Figure 4a). Moderate-to-severe PVL was uncommon in the entire series (4.7%); however, there was a significant over 3-fold increase in moderate-to-severe PVL risk with ACURATE neo implantation: (RR 3.06, 95%CI, [2.09, 4.49] P<0.00001; I2 = 0%) (Figure 4b) and corresponding incidence of 7.6% (96/1,256) and 2.3% (36/1,562) in ACURATE neo and SAPIEN 3 valves.

(6) 3.6. All-Cause Mortality

**Figure 5:**
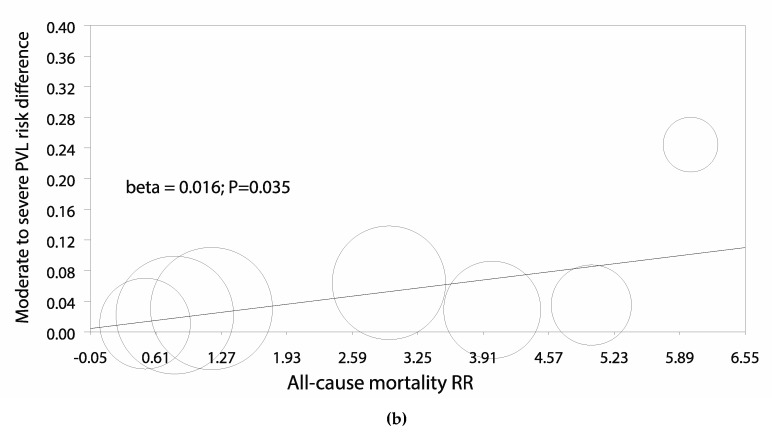
Individual and summary risk ratios with corresponding 95% confidence intervals for the comparison of ACURATE neo vs SAPIEN 3 in the analysis of (**a**) 30-day all-cause mortality; (**b**,**c**) meta regression analyses.

A random-effects meta-regression was fitted, counter-opposing all-cause mortality risk ratio against the risk difference of moderate-to-severe PVL; there was a trend for higher 30-day mortality rates with higher incidence of moderate-to-severe PVL (beta = 0.023; P = 0.093) (Figure 5b);

Should be replaced with:

A random-effects meta-regression was fitted, counter-opposing all-cause mortality risk ratio against the risk difference of moderate-to-severe PVL, showing higher 30-day mortality rates with higher incidence of moderate-to-severe PVL (beta = 0.016; P = 0.035) (Figure 5b);

(7) 4. Discussion

The abovementioned improvements seen in next-generation devices seem not to be the case with ACURATE neo; in the meta-analysis, we found 11.7% incidence of moderate-to-severe PVL in the ACURATE neo arm, nearly fourfold higher than in SAPIEN 3 and mild PVL in 45.5% cases, translating into 60% increased risk.

Should be replaced with:

The abovementioned improvements seen in next-generation devices seem not to be the case with ACURATE neo; in the meta-analysis, we found 7.6% incidence of moderate-to-severe PVL in the ACURATE neo arm, over 3-fold higher than in SAPIEN 3 and mild PVL in 45.5% cases, translating into 60% increased risk.

(8) 5. Conclusions

Moderate-to-severe PVL rates were, however, higher in ACURATE neo valve and were indirectly associated with increased 30-day all-cause mortality.

Should be replaced with:

Moderate-to-severe PVL rates were, however, higher in ACURATE neo valve and were associated with increased 30-day all-cause mortality.

The incorrect copying of the numerical data before statistical calculations does not affect the results presented in the paper other than what is stated in the conclusions. The authors apologize to the readers for any inconvenience caused by these changes. It is important to state that this correction does not affect our study’s results and involves no changes in the remaining data supporting our results. The original manuscript will remain online on the article webpage, with reference to this Correction.
